# OTUD1 exacerbates sepsis-associated encephalopathy by promoting HK2 mitochondrial release to drive microglia pyroptosis

**DOI:** 10.1186/s12974-025-03480-w

**Published:** 2025-06-11

**Authors:** Guoqing Jing, Hailong Gong, Han Wang, Jing Zuo, Die Wu, Huifan Liu, Xing Wang, Min Yuan, Yun Xia, Tongtong Du, Wanhong Liu, Xiaojing Wu, Xuemin Song

**Affiliations:** 1 Research Centre of Anesthesiology and Critical Care Medicine, Zhongnan Hospital of Wuhan University, Wuhan, Hubei Province China; 2 Department of Anesthesiology, Peking University People’s Hospital, Qingdao, China; 3 Department of Anesthesiology, Women and Children’s Hospital, Qingdao University, Qingdao, Shandong Province China; 4 Department of Anesthesiology, Renmin Hospital of Wuhan University, Wuhan, Hubei Province China; 5 Department of Immunology, School of Basic Medical Sciences, Wuhan University, Wuhan, Hubei Province China

**Keywords:** OTUD1, SAE, Microglia, Pyroptosis, HK2

## Abstract

**Background:**

Sepsis-associated encephalopathy (SAE), a life-threatening neurological complication of systemic infection, contributes substantially to sepsis-related mortality. Accumulating evidence demonstrates that microglia-driven neuroinflammation emerges as a central pathogenic mechanism underlying SAE. Here, we identify ovarian tumor deubiquitinase 1 (OTUD1) as a critical mediator of SAE pathogenesis. We demonstrate that OTUD1 promotes hexokinase 2 (HK2) dissociation from mitochondria via selective K63-linked deubiquitination, triggering microglia pyroptosis and neuroinflammation. Our findings address a key knowledge gap by elucidating the OTUD1-HK2 axis as a novel regulatory pathway in SAE, offering potential therapeutic targets to mitigate cognitive deficits in sepsis.

**Methods:**

Single-cell RNA sequencing was used to identify SAE-specific microglia subpopulations and analyze the expression of deubiquitinases within these subpopulations. OTUD1 knockout mice were generated to investigate the role of OTUD1 in SAE. Both wild-type and OTUD1 knockout mice were subjected to cecal ligation and puncture to induce SAE. In vitro, primary microglia and BV2 cells were treated with LPS and nigericin to simulate inflammatory conditions. Cognitive function of the mice was assessed through behavioral tests. Neuronal and synaptic damage were evaluated using HE and Nissl staining, as well as transmission electron microscopy. ELISA and qPCR were used to detect neuroinflammation. Western blot and immunofluorescence were employed to analyze protein expression. Molecular docking, 3D confocal microscopy, and co-immunoprecipitation were conducted to detect the interaction between OTUD1 and HK2. Finally, the correlation between OTUD1 and SAE was evaluated by analyzing clinical samples.

**Results:**

Through single-cell RNA seq and subpopulation analysis, we identified an SAE-associated microglia (SAM) subpopulation with high expression of pyroptosis-related genes. Deubiquitinase expression analysis showed significantly elevated OTUD1 expression in SAM. OTUD1 deficiency attenuated neural damage and cognitive dysfunction in SAE mice in vivo. Further experiments revealed that OTUD1 regulates pyroptosis in microglia, affecting the progression of SAE. Mechanistically, OTUD1 directly binds to the C-terminal domain of HK2 through its Ala-rich domain and selectively cleaves K63-linked polyubiquitin chains on HK2 to promote the dissociation of HK2 from mitochondria, thereby activating the NLRP3 inflammasome and pyroptosis.

**Conclusions:**

In SAE, OTUD1 deubiquitinates HK2, promoting its dissociation from mitochondria, which triggers microglia pyroptosis, leading to neuronal damage and cognitive impairment.

**Supplementary Information:**

The online version contains supplementary material available at 10.1186/s12974-025-03480-w.

## Introduction

Sepsis-associated encephalopathy (SAE) is a diffuse brain dysfunction that occurs in patients with sepsis without direct infection of the central nervous system (CNS). SAE presents with a range of neurological symptoms, including confusion, delirium, cognitive impairment, and even coma, which is a severe complication of sepsis with increased morbidity and mortality [1, 2]. While the exact mechanisms of SAE remain fully elucidated, glial cell-mediated neuroinflammation is a critical component in its pathogenesis [3, 4].

Microglia are the primary immune cells in the CNS. During sepsis-induced systemic inflammation, they can be rapidly activated and release various pro-inflammatory cytokines, exacerbating inflammation in the brain, leading to neuronal damage and resulting in symptoms such as cognitive impairment and altered consciousness in SAE patients [5, 6]. Microglia pyroptosis is crucial in the development of SAE. During sepsis, microglia detect pathogen-associated molecular patterns or damage associated molecular patterns, which triggers a priming signal upregulating NLRP3 and pro-IL-1β/IL-18. A second signal, driven by stress factors like potassium efflux or reactive oxygen species [7, 8]activates the NLRP3 inflammasome, leading to its assembly with ASC and pro-caspase-1. Caspase-1 is then activated, cleaving pro-IL-1β and pro-IL-18 into their active forms, and gasdermin D (GSDMD) to form membrane pores, inducing pyroptosis and releasing cytokines, worsening neuroinflammation, and contributing to brain injury in SAE [9, 10].

Recent studies have highlighted the pivotal role of mitochondrial dysfunction in NLRP3 inflammasome activation and pyroptosis. Hexokinase 2 (HK2), a glycolytic enzyme anchored to the outer mitochondrial membrane via voltage-dependent anion channel 1 (VDAC1), directly regulates NLRP3 inflammasome assembly. Under inflammatory stimuli (e.g., LPS), HK2 dissociates from VDAC1, triggering mitochondrial Ca²⁺ overload and mitochondrial DNA (mtDNA) release, which activates NLRP3 inflammasome [11, 12] and subsequent pyroptosis. However, the molecular mechanisms governing HK2 dissociation from mitochondria in microglia during sepsis remain unknown.

Mechanistic studies reveal that HK2 mitochondrial localization is regulated by K63-linked ubiquitination, which stabilizes its interaction with VDAC1^13^. This suggests that deubiquitinating enzymes (DUBs)-proteases removing ubiquitin chains-could modulate HK2’s inflammatory activity. Among DUBs, ovarian tumor deubiquitinase 1 (OTUD1) is a compelling candidate due to its established roles in inflammatory pathologies and specificity for K63-linked ubiquitin chains. OTUD1 promotes inflammation in myocardial injury by activating NF-κB signaling [13] but paradoxically suppresses colitis by deubiquitinating RIPK1^15^. highlighting its context-dependent functions. Critically, OTUD1’s involvement in neuroinflammation and its potential regulation of HK2-VDAC1 interactions have not been explored.

Using single-cell RNA sequencing (scRNA-seq) of hippocampal microglia in a murine SAE model, we identified an SAE-associated microglia (SAM) subpopulation enriched with pyroptotic markers and overexpressing OTUD1. This transcriptional signature, coupled with OTUD1’s known affinity for K63-linked substrates, prompted us to hypothesize that OTUD1 drives microglia pyroptosis in SAE by deubiquitinating HK2 to disrupt HK2-VDAC1 binding.

Through this study, we demonstrated that OTUD1 induced neuronal damage by promoting microglia pyroptosis, which led to cognitive dysfunction and the progression of SAE. Additionally, we revealed that OTUD1 facilitated the dissociation of HK2 from the mitochondrial by removing K63-linked polyubiquitin chains, activating the NLRP3 inflammasome and triggering pyroptosis. These findings highlight the crucial role of OTUD1 in neuroinflammation, suggesting that targeting OTUD1 could serve as a potential therapeutic approach for SAE.

## Materials and methods

### Animals and sepsis model

8-week-old male C57BL/6J mice were used, including wild-type (WT) mice from the Hubei Province Center and OTUD1^−/−^ mice from Cyagen Biosciences. Mice were housed at 22 °C, 50–60% humidity, on a 12-hour light/dark cycle with free access to food and water. Cecal ligation and puncture (CLP) is a classical model for inducing sepsis. Numerous studies have demonstrated that CLP also compromises the blood-brain barrier, induces neuroinflammation, leads to neuronal damage, and ultimately results in cognitive dysfunction [4, 14]. Therefore, in our in vivo experiments, CLP was utilized to establish a mouse model of SAE. Laparotomy was performed under pentobarbital anesthesia (50 mg/kg), with cecal ligation and puncture using a 21 G needle. Sham group mice underwent the same procedure without ligation and puncture. All animal care and experiments were performed according to the ethical regulations set by the Animal Experimentation Committee of Wuhan University (WP20250062).

### ScRNA-seq library construction and sequencing

The hippocampus was washed, dissociated, and assessed for cell count and viability using a fluorescence cell analyzer with AO/PI reagents. Cells were resuspended at 1 × 10⁶ cells/ml, and scRNA-seq libraries were prepared with the SeekOne^®^ Digital Droplet 3’ Library Kit. Cells were mixed with reverse transcription reagents, barcoded beads, and oil in a SeekOne^®^ chip to form emulsion droplets. After reverse transcription, cDNA was purified, amplified, and prepared for sequencing. Libraries were quality-checked and sequenced on the Illumina NovaSeq 6000 platform (PE150).

### ScRNA-seq data analysis

The single-cell matrix was processed in R using Seurat (V4.4.0) for dimensionality reduction, clustering, and quality control, filtering low-quality cells, and removing doublets with DoubletFinder (V2.0.4). Batch effects were corrected via CCA, and marker genes were identified with FindAllMarkers, annotated using SingleR and CellMarker 2.0. Differential gene expression was analyzed and visualized with Seurat, scRNAtoolVis, ggpubr, and ggplot2. KEGG and GO enrichment analyses were conducted using clusterProfiler. Microglia subpopulations were re-clustered, and marker genes were identified. Transcription factor analysis utilized pySCENIC, including GRNBoost, RcisTarget, and AUCell. Pseudotime analysis was performed with monocle3 and visualized with ClusterGVis.

### Immunofluorescence staining

For hippocampal tissue, multiple immunofluorescence staining based on tyramide signal amplification technology was performed on formalin-fixed, paraffin-embedded brain sections. After blocking with 5% BSA, paraffin brain slices were incubated overnight at 4 °C with anti-OTUD1 (1:200, ab122481, Abcam), NeuN (1:200, 94403, CST), GFAP (1:200, 3670, CST), GSDMD-NT (1:200, ab215203, Abcam), and Iba-1 (1:200, ab178846, Abcam) antibodies. The slices were then incubated with FITC/CY3/CY5-conjugated secondary antibodies (1:400, Abbkine) at 37 °C for 1 h. For primary microglia, cells were fixed with 4% paraformaldehyde for 30 min and then permeabilized with 0.5% Triton X-100 for 30 min. The blocking step was carried out with 5% BSA for 1 h, and then the cells were incubated with ASC (1:200, AG-25B-0006, Adipogen), OTUD1 (1:200, ab122481, Abcam), HK2(1:200, 22029-1-AP, Proteintech), VDAC1 (1:200, 66345-1-Ig, Proteintech) overnight at 4 °C. After washing, the cells were stained with FITC/CY3-conjugated secondary antibodies (1:400, Abbkine) for 1 h. DAPI was used to stain the nuclei.

### Novel object recognition test (NORT)

The NORT was conducted following the reported protocols [15, 16] with minor modifications, comprising three sequential phases. Initially, mice underwent a 3-day habituation period where they were positioned in a 20 × 30 × 30 cm arena facing a sidewall and permitted unrestricted exploration for 3 min daily. Subsequently, a familiarization trial was implemented 24 h post-habituation, during which two indistinguishable objects were symmetrically placed in the arena for 5 min of unrestricted investigation. Exploratory behavior was operationally defined as nasal proximity ≤ 2 cm to an object. Following this phase, one of the objects was replaced by a novel object with different shapes and colors after a 24-hour interval. Mice were reintroduced to the modified environment for a 5-minute test session, with their interactions recorded via behavioral analysis software. The preference index was calculated as the ratio of time spent investigating the novel object to total exploration time (novel + familiar objects).

### Morris water maze test (MWMT)

Mice underwent four days of training followed by a probe trial on the fifth day. The test used a circular pool (1.2 m diameter, 0.6 m height) and a hidden platform in the southwest quadrant. Mice had 60 s to find the platform, and their latency to reach it was recorded. On the fifth day, the platform was removed, and mice swam for 60 s. Platform crossings and time spent in the target quadrant were recorded.

### Histopathology

Nissl staining and HE staining were performed to evaluate neuronal damage and loss. After paraffin embedding and sectioning, brain tissues were stained with a 1% toluidine blue solution and stained with HE solution following standard procedures.

### Cell culture and treatment

Primary hippocampal microglia were isolated from neonatal WT and OTUD1^−/−^ mice (P1-2). Hippocampal tissues were digested with trypsin, filtered, and cultured in DMEM-F12 with 10% FBS (GIBCO) in poly-L-lysine-coated T75 flasks. Cells were maintained at 37 °C with 5% CO2, with medium changes every 3 days. Microglia were isolated by shaking after two weeks. BV2 and HT22 cell lines from Abcell (Beijing, China) were cultured in DMEM with 10% FBS under the same conditions. To activate the NLRP3 inflammasome, primary microglia and BV2 cells were treated with 500 ng/ml LPS (from E. coli O111:B4, Sigma-Aldrich, Cat# L2630) for 4 h, followed by 10 µM nigericin for 45 min. Based on our previous findings [17] and prior research [18]the LPS/nigericin-induced microglia pyroptosis model effectively recapitulates key pathophysiological mechanisms of SAE in vitro. LPS, a TLR4 agonist, mimics systemic sepsis-induced inflammation by priming the NLRP3 inflammasome through NF-κB activation, akin to the cytokine storm observed in SAE patients. Nigericin, acting as a canonical second signal, triggers K^+^ efflux to drive inflammasome assembly, mirroring sepsis-associated metabolic stressors (e.g., mitochondrial ROS and ion flux). The two-step protocol (LPS + nigericin), a well-established method for studying microglia pyroptosis in vitro, ultimately induces caspase-1/GSDMD cleavage and IL-1β/IL-18 release.

### Establishment of microglia-conditioned medium

Primary microglia were treated with DMSO or LPS/nigericin. Then, the medium was replaced, and cells were cultured for 24 h. The supernatant was collected and centrifuged at 1000 rpm for 5 min. The medium for HT22 cells was replaced with the collected conditioned medium, and the cells were cultured for 12 h.

### Plasmids and SiRNA transfection

SiRNA was obtained from Tsingke Biotechnology Co., Ltd (Nanning, China) and transfected into BV2 cells. The sequences used were as follows: siOTUD1 sense: 5’-CCAGAACGGCGAAGGCGAATT-3’, and negative control siRNA (siNC) sense: 5’-UUCUCCGAACGUGUCACGUTT-3’. The plasmid CMV-ER-LAR-GECO1 was a gift from Pro. Song Han. Flag-OTUD1, Myc-HK2, Flag-OTUD1-C320S, Flag-OTUD1-ΔAla, Flag-OTUD1-Δlinker, Flag-OTUD1-ΔOTU, Flag-OTUD1-ΔUIM, Myc-HK2-ΔN, Myc-HK2-ΔC, HA-Ub-WT, HA-Ub-K63, HA-Ub-K48 plasmids were purchased from Miaoling Biotechnology (Wuhan, China).

### Cell viability

HT22 cells were seeded in a 96-well plate, cultured for 24 h, then treated with microglia-conditioned medium for 12 h. Cell viability was assessed by CCK-8 reagent (Biosharp).

### Confocal microscopy analysis

Confocal imaging was conducted using a Leica TCS SP8 microscope, with all images acquired in serial frame acquisition mode. XYZ-series were captured at a raster size of 1024 × 1024 in the XY plane, with a Z-step interval of 0.2 μm between optical sections. Three-dimensional images and projections from the z-stack were created and processed using Leica Application Suite X software.

### Real-time quantitative polymerase chain reaction (RT-qPCR)

Total RNA was isolated from primary microglia using TRIZOL Reagent (Invitrogen). The extracted RNA was reverse transcribed into complementary DNA using RT-qPCR Fast Master Mix (Vazyme, China). Specific primers used for RT-qPCR assays were 5′-GGCATGGATCTCAAAGACAACC-3′, 5′-CAGGTATATGGGCTCATACCAG-3′ for TNF-α; 5′-CATGTTCTCTGGGAAATCGTGG-3′, 5′-GTACTCCAGGTAGCTATGGTAC-3′ for IL-6; 5′-GAAATGCCACCTTTTGACAGTG-3′, 5′-TGGATGCTCTCATCAGGACAG-3′ for IL-1β; 5′-CAGGCTGTCTTTTGTCAACGA-3′, 5′-GACTCTTGCGTCAACTTCAAGG-3′ for IL-18; 5′-GATATCGCTGCGCTGGTCG-3′, 5′- CATTCCCACCATCACACCCT-3′ for β-actin.

### Transmission electron microscopy (TEM)

For each experimental group, TEM images were systematically captured from the hippocampal CA3 region of 3 animals per group. 5 ultrathin sections (70 nm thickness) per animal were analyzed at 15,000× magnification. A total of 100–120 synaptic structures per group were randomly selected across sections (Wuhan Luochuang Biotechnology CO., Ltd). Postsynaptic density thickness and synaptic cleft width were measured using ImageJ software. Measurements were performed by two blinded investigators to ensure reproducibility.

### Western blot analysis

Total protein was extracted from hippocampus or cells using RIPA lysis buffer with protease inhibitors. Equal amounts of protein were separated by SDS-PAGE, transferred to PVDF membranes, blocked with 5% milk, and incubated with primary antibodies including OTUD1 (ab122481, Abcam), NLRP3 (15101, CST), GSDMD (39754, CST), Caspase-1 (AG-20B-0042, Adipogen), ASC (AG-25B-0006, Adipogen), Iba-1 (ab178846, Abcam), HK2 (22029-1-AP, Proteintech), VDAC1 (10866-1-AP, Proteintech), HA (51064-2-AP, Proteintech), Flag (20543-1-AP, Proteintech), Myc (60003-2-Ig, Proteintech), PSD95 (3409, CST), SYP (AF0257, Affinity), and β-actin (HRP-60008, Proteintech). After washing, membranes were incubated with HRP-conjugated secondary antibodies, washed again, and visualized using chemiluminescence.

### Co-immunoprecipitation

Cells were lysed on ice for 30 min using NP-40 lysis buffer. After centrifuging the lysates at 12,000 rpm for 15 min, the supernatant was collected and incubated with the target antibody at 4 °C with continuous rotation overnight. Protein A/G magnetic beads (HY-K0202, MCE) were added the next day, followed by further incubation with rotation for 4 h. The magnetic beads were washed thrice with wash buffer. Then SDS-PAGE sample buffer was added, and the mixture was heated at 95 °C for 10 min. The co-immunoprecipitated proteins were subsequently analyzed by western blot.

### ELISA

TNF-α, IL-6, IL-18, and IL-1β concentrations were measured using an ELISA kit (Beijing 4 A Biotech Co., Ltd) following the manufacturer’s guidelines.

### Statistical analysis

Data analysis and graphing were performed using GraphPad Prism 8.3.0. Results are shown as mean ± SEM. MWMT training was analyzed with two-way ANOVA for repeated measures and Bonferroni correction. Multiple group comparisons used two-way ANOVA with Bonferroni post-hoc tests. Statistical significance was set at *p* < 0.05. Pearson’s correlation analysis was employed to assess co-expression relationships between OTUD1 mRNA levels and cytokine concentrations.

## Results

### ScRNA-seq identifies SAM in the hippocampus of SAE mice

**Fig. 1:**
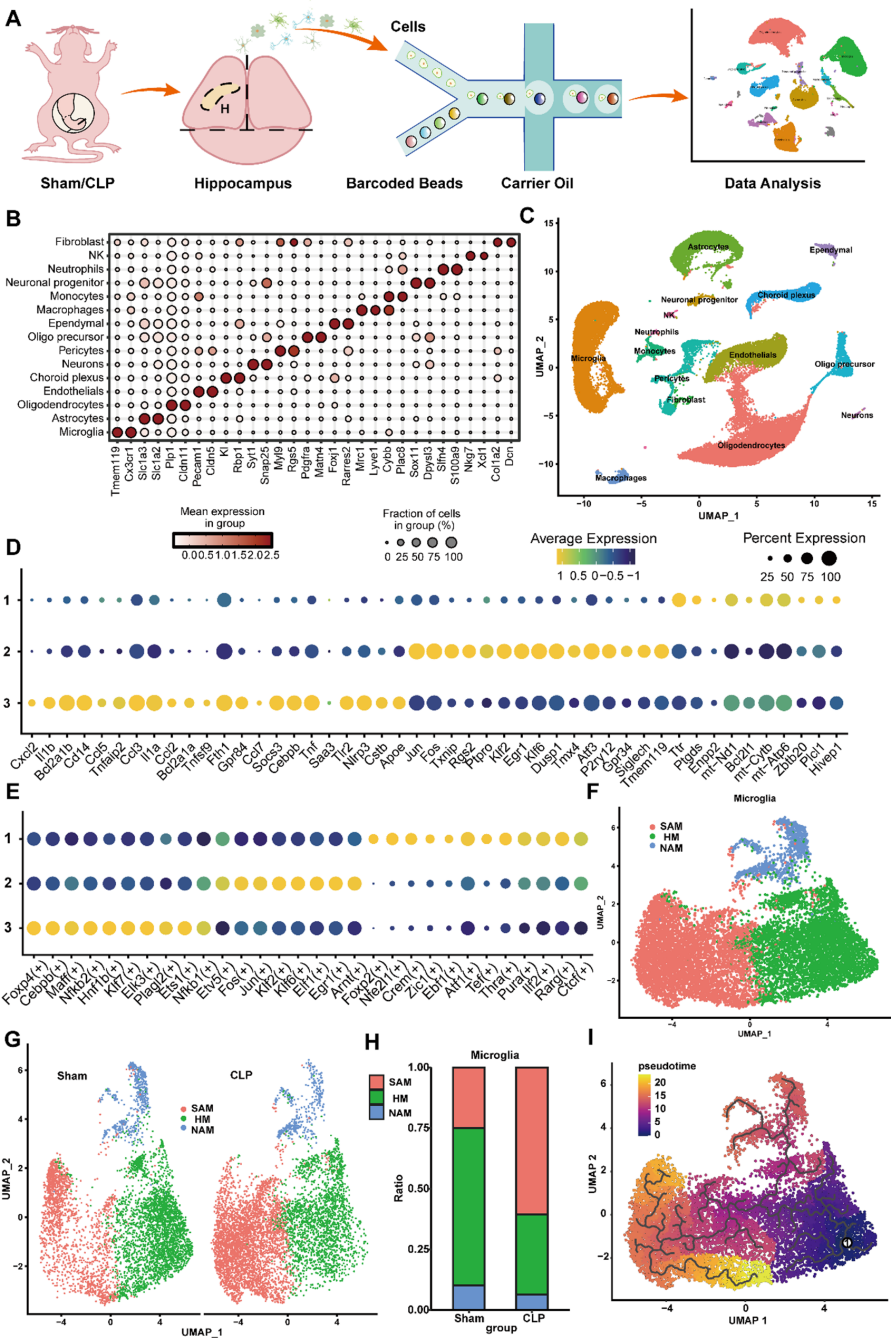
ScRNA-seq identifies SAM in the hippocampus of mice. (**A**) Experimental design for scRNA-seq (*n* = 2). (**B**) Identification of cell types in hippocampal tissue based on marker genes. (**C**) UMAP plot showing clusters and annotations of cells identified in hippocampus. (**D**) Dot plot displaying signature markers of each microglial subpopulation. (**E**) Dot plot exhibiting activated TFs for each microglial subpopulation. (**F**) UMAP plot of the microglial subpopulation. (**G**) UMAP plot demonstrating the distribution of microglial subpopulations across different groups. (**H**) Stacked bar graph showing the proportions of microglial subpopulations across different groups. (**I**) Pseudo-time graph projection of microglia illustrating the transition from HM (blue) to SAM (yellow).

To characterize brain cell changes in septic mice, we conducted scRNA-seq on hippocampal cells from CLP and sham-operated mice (Fig. 1A). Unsupervised clustering and gene marker analysis identified 15 cell types, with microglia, oligodendrocytes, and astrocytes as the most abundant (Figs. 1B–C, Supplementary Table S1). Given the study’s focused investigation on functional dynamics of microglia, we employed scRNA-seq rather than single-nucleus RNA sequencing (snRNA-seq) as the primary analytical approach. This methodological selection prioritized sensitivity in detecting dynamic changes and data comprehensiveness for microglial profiling. However, due to the inherent limitations in somatic capture efficiency for neuronal cells within scRNA-seq workflows, the resulting dataset exhibited comparatively sparse neuronal cell populations. We then identified three microglia subpopulations based on their gene expression patterns (Fig. 1D & Supplementary Table S2). Cluster 1 expressed genes like *Ttr* and *Zbtb20* linked to nervous system homeostasis. Cluster 2 highly expressed microglia homeostatic signature genes such as *P2ry12* and *Tmem119*. Cluster 3 had elevated expression of inflammation-related genes (*Il1b*, *Tnf*) and monocyte chemoattractants (*Ccl5*, *Ccl3*), indicating a pro-inflammatory role. SCENIC analysis revealed active inflammation-related transcription factors (TFs) in in Cluster 3, such as *Foxp4*, *Cebpb*, and *Nf-κb2* (Fig. 1E). Based on the gene expression and transcriptional regulation analysis within different subpopulations, we named Cluster 1, 2, and 3 as neural regulation-associated microglia (NAM), homeostatic microglia (HM), and SAM, respectively (Fig. 1F). In sham-operated mice, HM dominated, with fewer SAM, while CLP mice showed a significant increase in SAM and a decrease in HM, with stable NAM proportions (Fig. 1G-H). Pseudotime analysis confirmed a trajectory from HM to SAM, reflecting a transcriptional shift during sepsis (Fig. 1I). These findings highlight SAM as key microglia populations involved in SAE development.

### OTUD1 and pyroptosis-related gene expression is increased in SAM

**Fig. 2:**
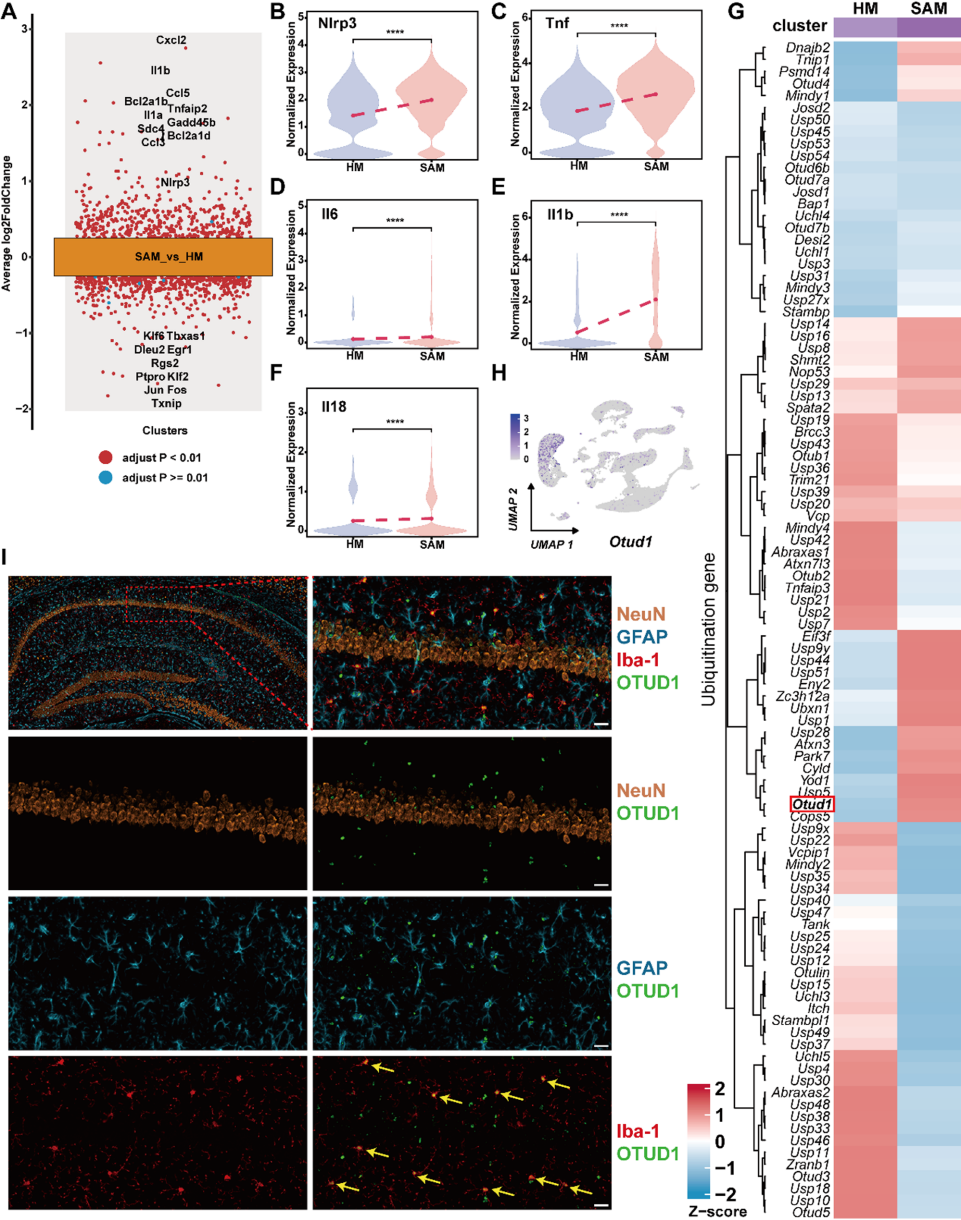
OTUD1 and pyroptosis-related genes expression is increased in SAM. (**A**) Volcano plot showing the differentially expressed genes between SAM and HM. (**B-F**) Violin plots displaying the expression of *Nlrp3*, *Tnf*, *Il6*, *Il1b*, *Il18* between SAM and HM. (**G**) Heatmap exhibiting the expression of deubiquitinases in microglial subpopulations. (**H**) UMAP plot showing the expression of OTUD1 in different cell types of the hippocampus. (**I**) The colocalization of OTUD1 with neurons, microglia, and astrocytes was detected by multiplex immunofluorescence in WT mice (*n* = 5). Yellow arrows indicate microglia showing co-localization of OTUD1 and IBA1. Scale bars, 20 μm. ****p < 0.0001 vs. HM.

To further explore the mechanisms and key molecules involved in the pathogenesis of SAE, we conducted a differential gene expression analysis between SAM and HM (Fig. 2A). The results show significantly higher IL-1β levels in SAM compared to HM. The formation of membrane pores during NLRP3 inflammasome activation-mediated pyroptosis is a key pathway for releasing IL-1β from within the cell. Therefore, we examined the expression of pyroptosis-related genes and found that *Nlrp3*, *Tnf*, *Il1b*, and *Il18* were significantly upregulated in SAM (Fig. 2B-F), suggesting that pyroptosis played a crucial role in SAE. Given the importance of DUBs in immune regulation during sepsis, we analyzed their expression across different microglia subpopulations. As shown in Fig. 2C, compared to HM, over a dozen DUBs were upregulated in SAM, with OTUD1 showing one of the most pronounced changes among them. To verify whether OTUD1 primarily functions in microglia, we first analyzed the cell-specific expression of OTUD1. Intriguingly, scRNA-seq analysis identified predominant expression of OTUD1 in microglia within the mouse hippocampus (Fig. 2H). Furthermore, multiplex immunofluorescence staining in WT mice revealed marked co-localization of OTUD1 with the microglial marker IBA-1, but minimal overlap with astrocytic (GFAP) or neuronal (NeuN) markers (Fig. 2I). These findings collectively establish that OTUD1 exhibits microglia-specific expression in the hippocampal region.

### OTUD1 knockout alleviates cognitive dysfunction in SAE mice

**Fig. 3:**
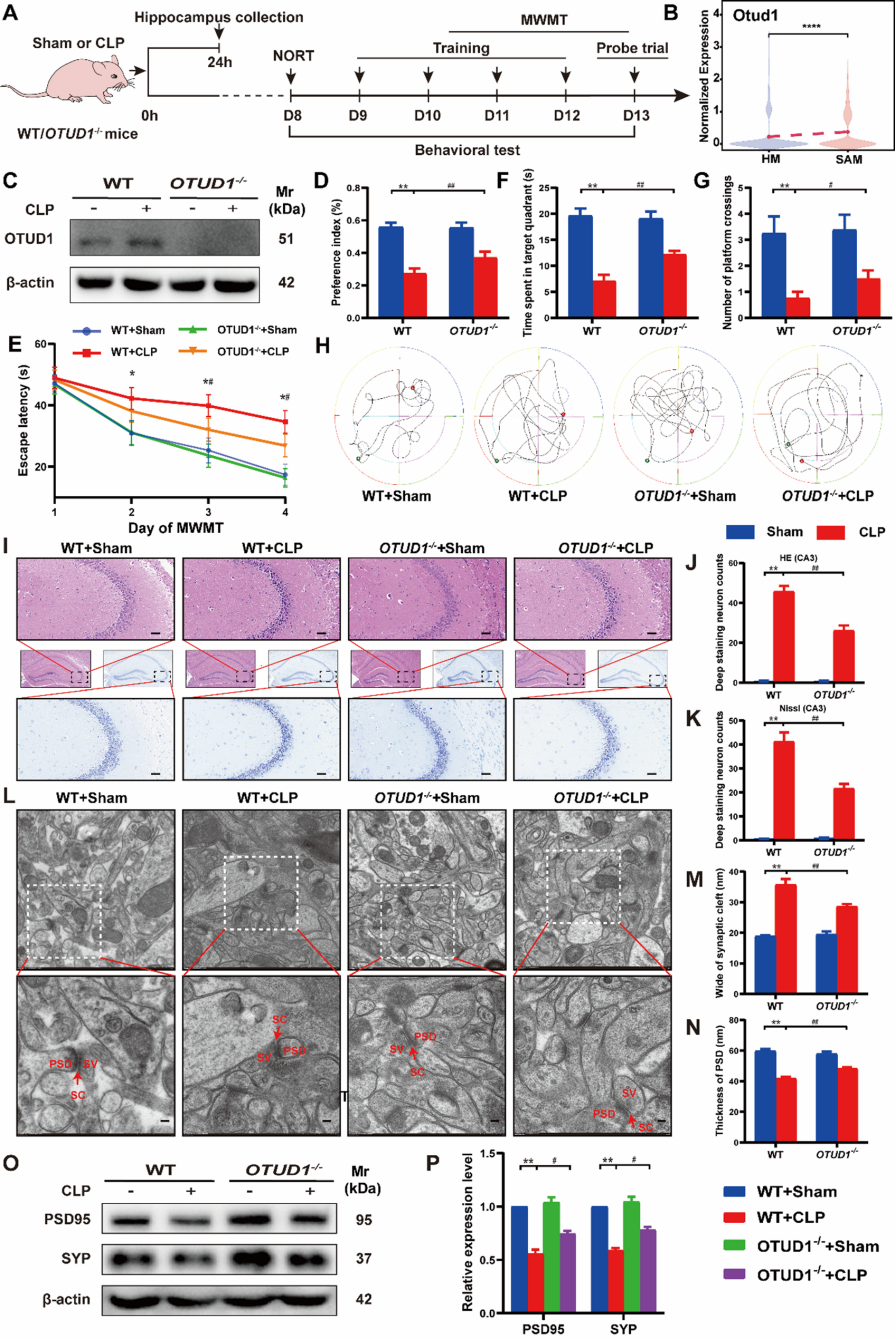
OTUD1 knockout alleviates cognitive dysfunction in SAE mice by reducing neuronal and synaptic damage. (**A**) Experimental design for animals in vivo. (**B**) Violin plots displaying the expression of *Otud1* in microglial subclusters. (**C**) OTUD1 expression in hippocampus was analyzed by western blot (*n* = 3/group). (**D**) The preference index of the four groups of mice in the NORT (*n* = 8/group). (**E**) Escape latency, (**F**)time spent in the target quadrant, and (**G**) numbers of crossings through the platform were recorded in each group (*n* = 8/group). (**H**) Trajectory plot of spatial exploration in MWMT. (**I-K**) Neuronal damage of the hippocampal region was assessed by HE staining and Nissl staining (*n* = 5/group). Scale bar, 50 μm. (**L**) Synaptic structural changes were observed through TEM. SV, Synaptic vesicles; SC, Synaptic cleft; PSD, Postsynaptic density. Scale bar, 100 nm. (**M-N**) Image analysis of the thickness of PSD and the width of the synaptic cleft (*n* = 3/group). (**O-P**) The levels of PSD95 and SYP in hippocampus were measured by western blot (*n* = 3/group). *p < 0.05, **p < 0.01 vs. WT + Sham group; ^#^*p* < 0.05, ^##^*p* < 0.01 vs. WT + CLP group.

To further explore the physiological role of OTUD1 in SAE, we generated OTUD1 knockout mice. We first examined the expression changes of OTUD1 in SAE and validated the knockout efficiency in OTUD1^−/−^ mice. Consistent with the mRNA level changes observed in scRNA-seq (Fig. 3B), western blot showed that OTUD1 protein level was up-regulated during sepsis (Fig. 3C), suggesting that elevated OTUD1 expression was associated with SAE. In addition, western blot analysis of hippocampus lysates demonstrated complete loss of OTUD1 protein in knockout mice. Cognitive dysfunction is the most prominent clinical manifestation of SAE (Fig. 3C). We then performed NORT and MWMT 7 days post-CLP to evaluate OTUD1’s role in cognition. In NORT, the preference index of CLP mice was significantly lower than that of the sham mice, which improved with OTUD1 knockout (Fig. 3D). In the MWMT navigation test, there was no difference in escape latency among the groups on the first day. On the second day, the CLP mice showed a longer latency compared to the non-surgery mice. Additionally, the escape latency of OTUD1^−/−^ septic mice on day 3 and 4 was shorter compared to the WT septic mice (Fig. 3E). Spatial exploration test showed CLP mice spent less time in the target quadrant and crossed the platform fewer times, but these deficits were partially rescued in OTUD1^−/−^ mice (Figs. 3F–H). Moreover, OTUD1 deletion alone had no significant cognitive effects. The behavioral experiments indicated that OTUD1 knockout could significantly mitigate cognitive impairment in CLP-induced SAE mice.

### OTUD1 deficiency alleviates neuronal and synaptic damage in SAE mice

The hippocampus is a key region in the brain responsible for learning and memory [19, 20]. In SAE, neuronal damage in the hippocampus directly affects these cognitive functions, impairing learning and memory abilities [21, 22]. As shown in Fig. 3I-K, CLP-induced SAE mice exhibited extensive neuronal damage in the hippocampus, particularly in the CA3 region, characterized by pyknotic nuclei and cytoplasmic atrophy. Compared with CLP group, more orderly neurons and fewer deeply stained cells were observed in OTUD1^−/−^ SAE mice, indicating a reduction in neuronal damage. We further examined the synaptic ultrastructural pathological changes in CA3 region via a TEM and the results showed blurred synaptic structures, swollen presynaptic terminals, thinner postsynaptic densities, and widened synaptic clefts in SAE mice. While OTUD1^−/−^ SAE mice exhibited improved synaptic integrity and less structural damage, with thicker postsynaptic densities and narrower synaptic clefts (Fig. 3L-N). Meanwhile, western blot analysis showed that the expression of two important presynaptic and postsynaptic proteins, postsynaptic density 95 (PSD95) and synaptophysin (SYP), were significantly downregulated in mice undergoing CLP surgery and higher levels of PSD95 and SYP were detected in septic mice with OTUD1 deletion (Fig. 3O-P). These data indicate that OTUD1 deficiency ameliorates SAE-induced cognitive deficits, potentially through attenuating hippocampal neuronal injury and synaptic loss while upregulating synaptic protein expression.

### OTUD1 deficiency reduces pyroptosis of microglia in vivo

**Fig. 4:**
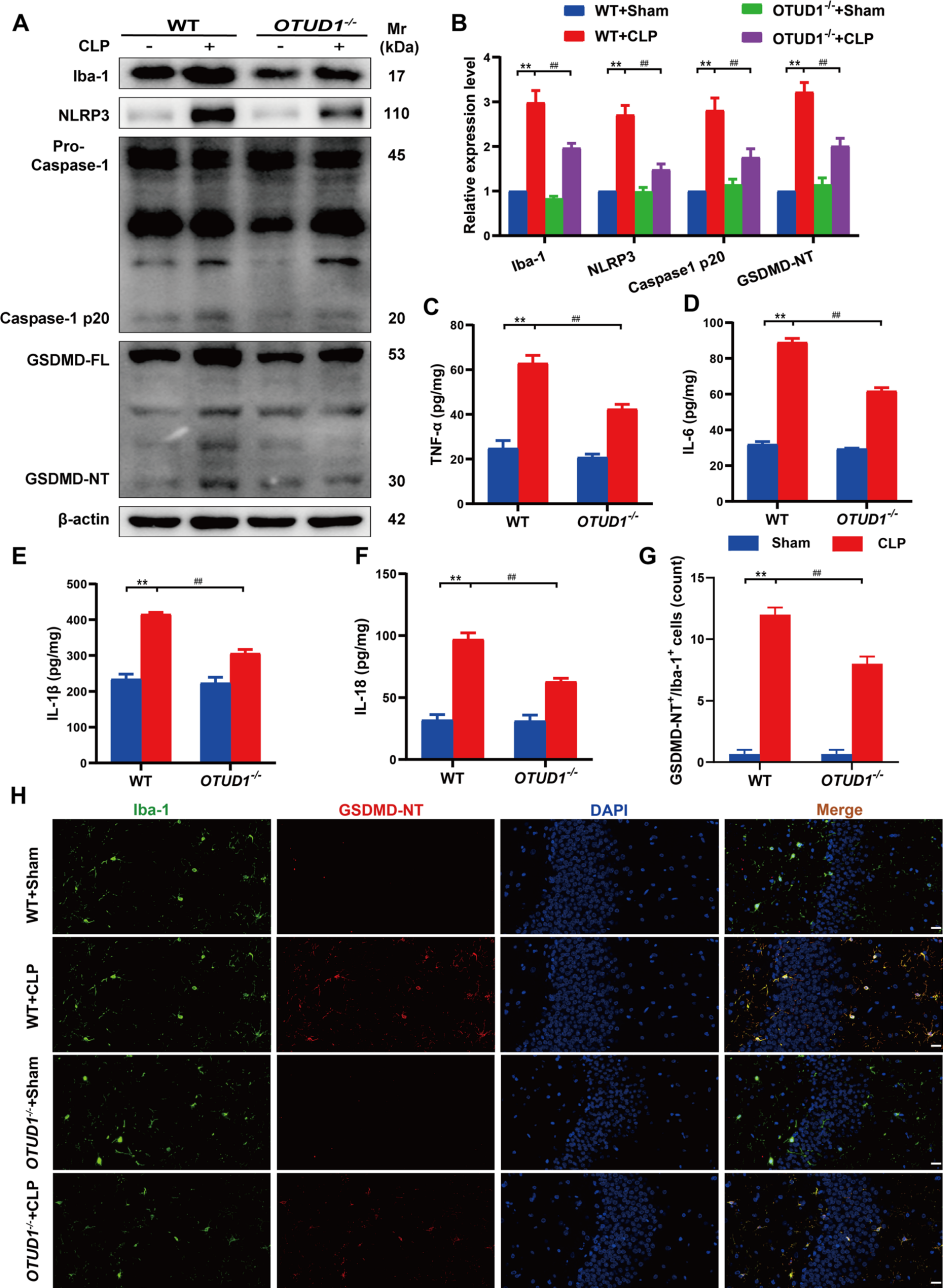
OTUD1 deficiency reduces pyroptosis of microglia in vivo.**(A-B)** The levels of Iba-1, NLRP3, Caspase-1 P20, and GSDMD-NT in the hippocampus were measured by western blot (*n* = 3/group). **(C-F)** TNF-α, IL-6, IL-1β and IL-18 were measured in the hippocampus by ELISA (*n* = 3/group). **(G-H) **Numbers of GSDMD-NT/Iba-1 positive cells in the hippocampus were measured by double immunofluorescence staining (*n* = 5/group). Scale bar, 20 μm. **p<0.01 vs. WT+Sham group, ^##^*p*<0.01vs. WT+CLP group.

Our previous studies have shown that microglia pyroptosis-mediated neuroinflammation is a potential accelerator of nerve injury, as well as learning and memory decline in SAE [17]. Microglia are also considered the main cells where pyroptosis occurs in CNS [23]. Therefore, we examined whether OTUD1-induced neural damage is associated with microglia pyroptosis. As shown in Fig. 4A-B, CLP surgery significantly increased the level of Iba-1(microglia activation marker), while OTUD1 knockout reduced its expression in the hippocampus of SAE mice, indicating decreased microglia activation. OTUD1 deficiency also reduced pyroptosis-related proteins, including NLRP3, Caspase-1 P20, and GSDMD-NT in SAE mice. Meanwhile, inflammatory cytokines (IL-1β, IL-18, TNF-α, and IL-6) elevated by CLP were effectively suppressed by OTUD1 knockout (Fig. 4C-F). Immunofluorescence also confirmed that pyroptosis occurred mainly in microglia, as indicated by increased GSDMD-NT/Iba-1 double-positive cells, which were reduced after OTUD1 deletion (Fig. 4G-H). Hence, we conclude that the absence of OTUD1 significantly reduced the pyroptosis in microglia in the hippocampus of SAE mice.

### OTUD1 deficiency reduces pyroptosis of microglia in vitro

**Fig. 5:**
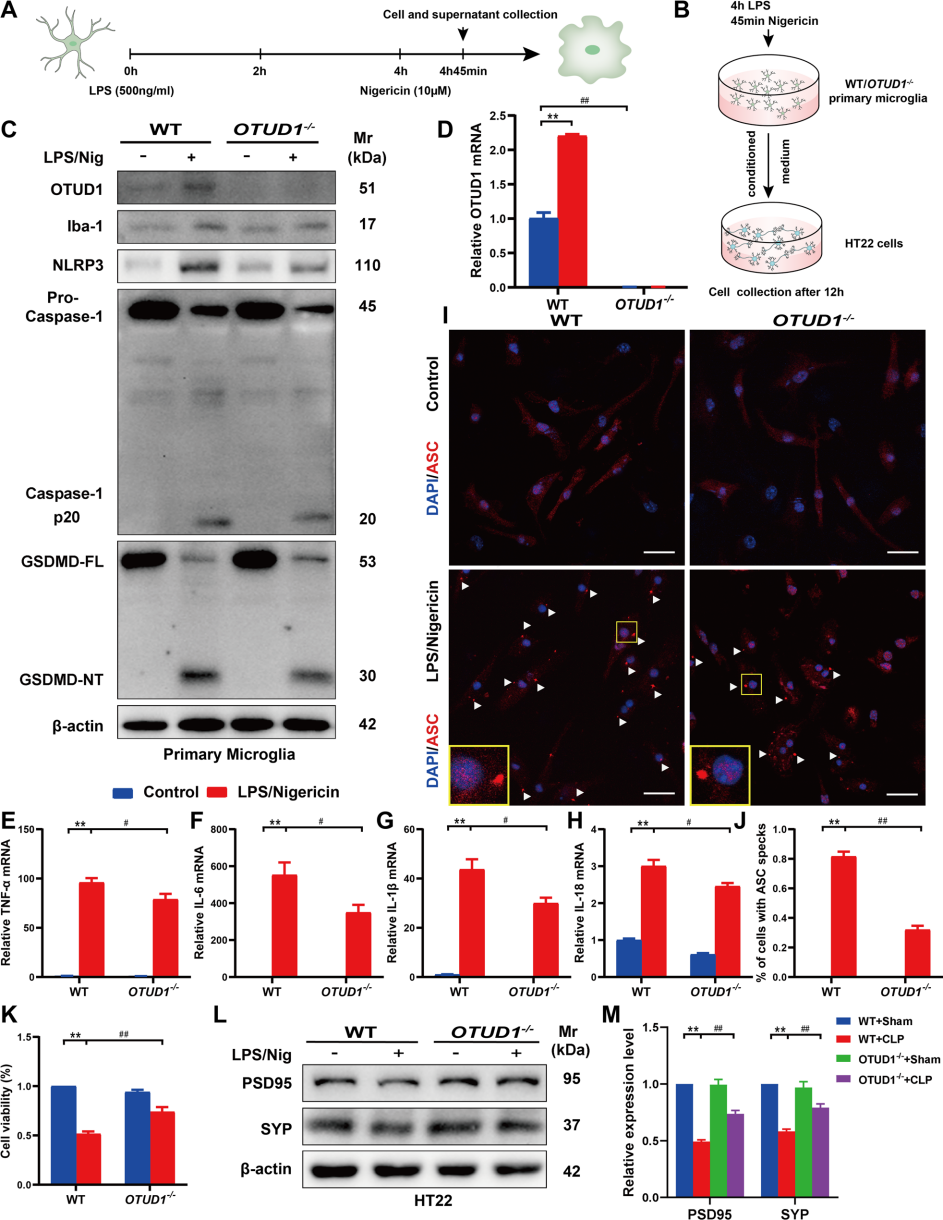
OTUD1 deficiency reduces pyroptosis of microglia in vitro. (A-B) Experimental design for cells in vitro. (C) The protein levels of OTUD1, Iba-1, NLRP3, Caspase-1 P20, and GSDMD-NT in primary microglia were measured by western blot (*n* = 3/group). (D-H) The mRNA levels of OTUD1, TNF-α, IL-6, IL-1β and IL-18 in primary microglia were measured by qPCR (*n* = 3/group). (I-J) ASC oligomerization in primary microglia were detected by immunofluorescence. Scale bar, 25 μm. (K) Cell viability of HT22 was examined by CCK8 (*n* = 5/group). (L-M) Synapse-related proteins PSD95 and SYP in HT22 cells were measured by western blot (*n* = 3/group). *p < 0.05, **p < 0.01 vs. Control group; #*p* < 0.05, ##*p* < 0.01 vs. LPS/Nigericin group.

Subsequently, we validated this conclusion using LPS/nigericin-treated primary microglia in vitro (Fig. 5A-B). OTUD1 expression, both mRNA and protein, was upregulated following LPS/nigericin stimulation (Fig. 5C-D & S1A). LPS/nigericin increased Iba-1, NLRP3, Caspase-1 P20, and GSDMD-NT levels, which were reduced by OTUD1 knockout (Fig. 5C & S1A). Additionally, qPCR results suggested that IL-1β, IL-18, TNF-α, and IL-6 were all increased after LPS/nigericin stimulation, and the pro-inflammatory cytokines were obviously decreased following OTUD1 knockout (Fig. 5E-H). The oligomerization of ASC in the cytoplasm is a key marker of pyroptosis, and the extent of pyroptosis can be assessed by detecting the number of ASC specks [24, 25]. Immunofluorescence results showed that LPS/nigericin significantly increased ASC oligomerization, as evidenced by an increase in the number of ASC specks, while OTUD1 deficiency markedly reduced ASC recruitment and speck formation (Fig. 5I-J). To more realistically simulate the effect of microglia pyroptosis on hippocampal neurons, we verified the in vivo findings through cell interaction experiments. The conditioned medium from LPS/nigericin-treated microglia significantly reduced cell viability and the expression of PSD95 and SYP in HT22 cells. Consistent with the in vitro results, cell viability and synapse-associated protein levels partially restored with OTUD1 deletion (Fig. 5K-M). Moreover, parallel experiments in BV2 cells yielded results concordant with those from primary microglia. The expressions of OTUD1 and pyroptosis-related proteins (NLRP3, Caspase-1 P20, and GSDMD-N) were enhanced in the LPS/nigericin group (Figure S1B-C), as well as the secretion of inflammatory cytokines (Figure S1D-F). While, OTUD1 knockdown prevented LPS/nigericin-induced BV2 pyroptosis and inflammatory cytokine release. In conclusion, OTUD1 deficiency can alleviate microglia pyroptosis in vivo and in vitro.

### OTUD1 promotes pyroptosis by facilitating the release of HK2 from mitochondria

**Fig. 6:**
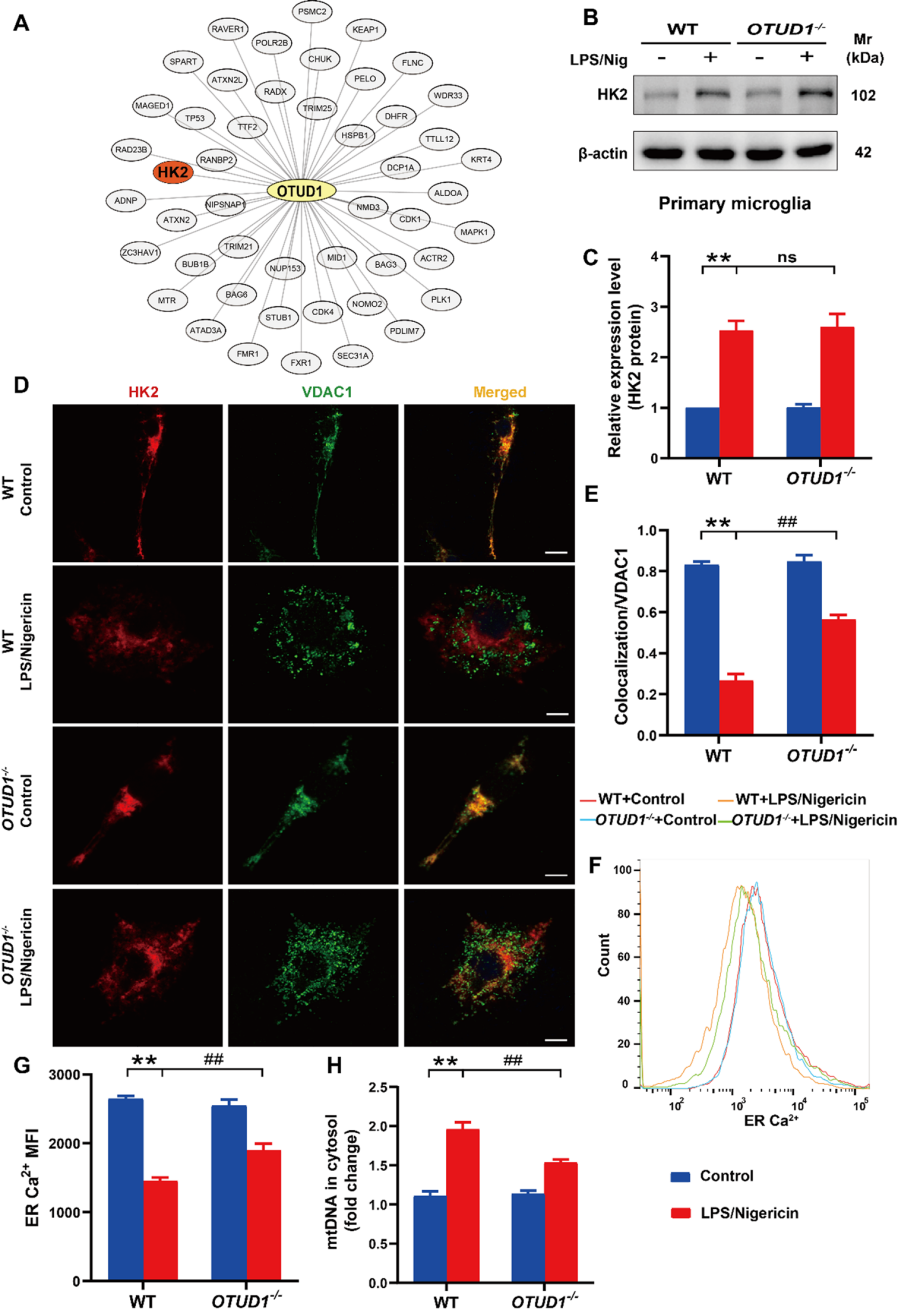
OTUD1 promotes pyroptosis by facilitating the release of HK2 from mitochondria. (**A**) Potential networks of interacting proteins of OTUD1 revealed by PPI String software. (**B-C**) The expression of HK2 in primary microglia were measured by western blot (*n* = 3/group). (**D-E**) The colocalization of HK2 and VDAC1 was analyzed by immunofluorescence (*n* = 3/group). Scale bar, 8 μm. (**F-G**) ER Ca^2+^ of primary microglia located by ER-targeted plasmid CMV-ER-LAR-GECO1 were observed by flow cytometry (*n* = 3/group). (**H**) The content of cytosolic mtDNA was detected by qPCR (*n* = 3/group). **p < 0.01 vs. Control group; ^##^*p* < 0.01 vs. LPS/Nigericin group.

Recent studies have highlighted the release of HK2 from mitochondria as a key feature of NLRP3 inflammasome activation [11, 26]. Based on the mass spectrometry results [27]we constructed a protein-protein interaction (PPI) network for OTUD1 and found HK2 may be a potential interacting protein of OTUD1 (Fig. 6A). To investigate the role of HK2 in OTUD1-regulated pyroptosis in microglia, we examined the expression of HK2 and found LPS/nigericin increased HK2 expression, but the absence of OTUD1 did not alter HK2 protein levels following LPS/nigericin treatment (Fig. 6B-C). We then explored the localization of HK2 in mitochondria, which associates with the outer mitochondrial membrane by interacting with VDAC1. Immunofluorescence microscopy revealed prominent colocalization of VDAC1 and HK2 in the resting state, and LPS/nigericin caused the release of HK2 from the mitochondria, manifested by a significant reduction in the colocalization of HK2 with VDAC1. However, the absence of OTUD1 enhanced the binding between HK2 and the mitochondrial outer membrane protein VDAC1 (Fig. 6D-E). This suggested that OTUD1 deletion reduced the dissociation of HK2 from the mitochondria. HK2 dissociation from VDAC1 can trigger the activation of inositol triphosphate receptors on the ER, releasing Ca^2+^ from the ER. The influx of Ca^2+^ into mitochondria induce the oligomerization of VDAC1, forming large pores that allow mtDNA to exit the mitochondria, activating the NLRP3 inflammasome [11]. We subsequently explored the impact of HK2 dissociation from the mitochondria on ER Ca^2+^ and mtDNA levels. Using the ER-targeted plasmid CMV-ER-LAR-GECO1, a highly sensitive indicator designed to monitor Ca^2+^ dynamics within the ER [28]we observed a noticeable reduction in ER Ca^2+^ levels following LPS/nigericin treatment. However, this reduction was alleviated in OTUD1^−/−^ primary microglia (Fig. 6F-G). The mtDNA in microglia subjected to LPS/nigericin was significantly enriched compared to the control group, while the cytosolic mtDNA content was markedly reduced upon OTUD1 deletion (Fig. 6H). These findings indicate that OTUD1 facilitates NLRP3 inflammasome activation and pyroptosis by regulating HK2 translocation, which in turn affects ER Ca^2+^ efflux and mtDNA release.

### OTUD1 interacts with HK2

**Fig. 7:**
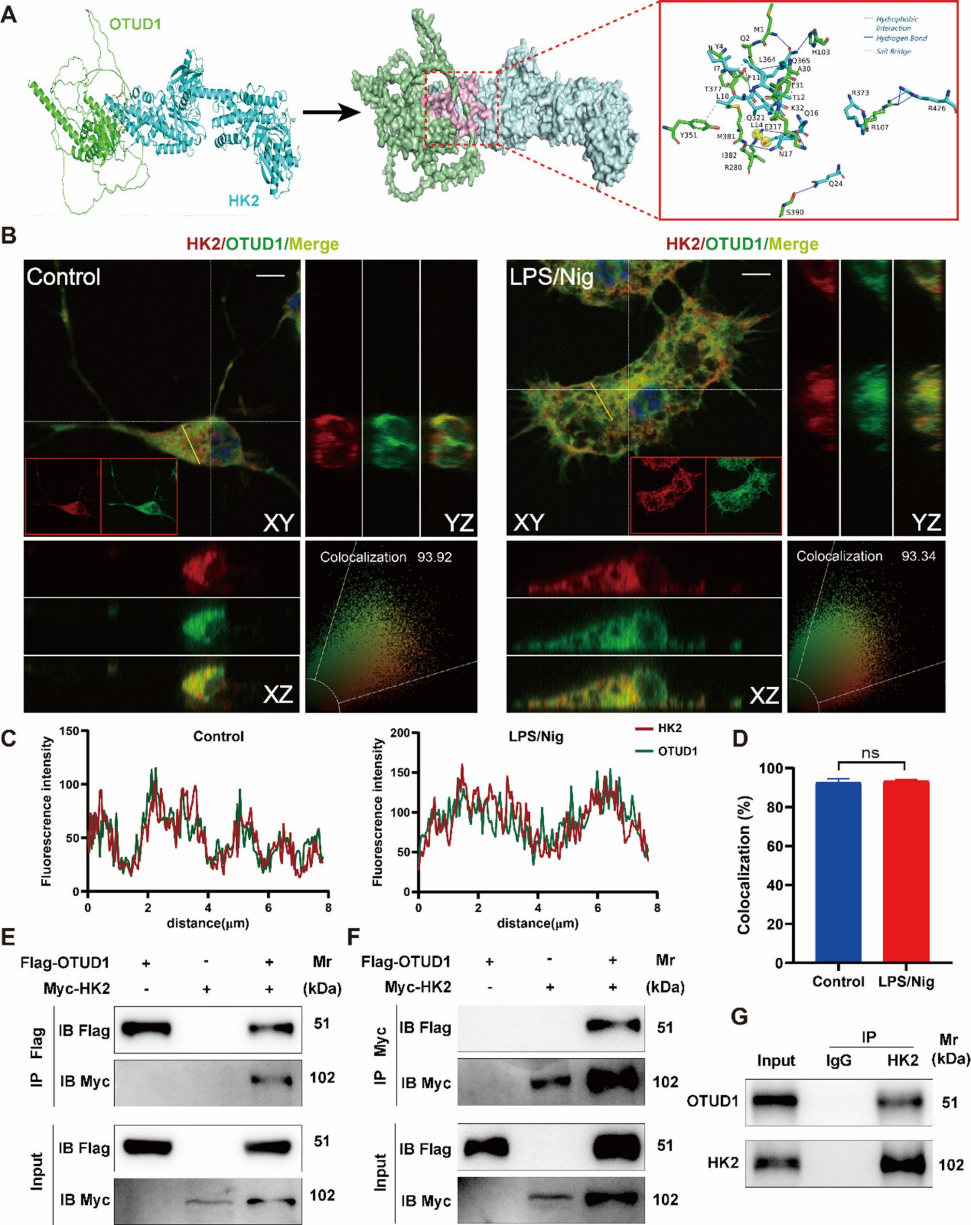
OTUD1 interacts with HK2. (**A**) Molecular docking predicts the possibility and binding sites of the interaction between OTUD1 and HK2. Scale bar, 5 μm. (**B-D**) 3D confocal imaging to detect the colocalization of OTUD1 and HK2 (*n* = 5/group). (**E-F**) Transfection of Flag-OTUD1 and Myc-HK2 plasmids into 293T cells, followed by exogenous Co-IP to detect the interaction between OTUD1 and HK2 (*n* = 3/group). (**G**) The interaction between OTUD1 and HK2 in BV2 cells was evaluated by endogenous Co-IP (*n* = 3/group).

To characterize the mechanism by which OTUD1 regulates the mitochondrial translocation of HK2, we used the HDOCK docking tool to predict the interaction between OTUD1 and HK2 firstly. Through this method, we obtained a predicted binding model of OTUD1 and HK2, revealing 6 hydrophobic interactions, 14 hydrogen bonds, and 1 salt bridge between the two proteins (Fig. 7A). The results of fluorescent colocalization and co-immunoprecipitation (Co-IP) subsequently verified the interaction between OTUD1 and HK2. Through three-dimensional confocal reconstruction, we not only observed the transformation of primary microglia from a small-bodied, ramified resting state to an enlarged, amoeboid-shaped activated state but also detected significant colocalization of OTUD1 and HK2 in terms of spatial distribution in microglia whether with or without LPS/nigericin stimulation (Fig. 7B-D). We transfected Flag-OTUD1 and Myc-HK2 plasmids into HEK-293T cells, and showed that OTUD1 interacts with HK2 following Co-IP assay (Fig. 7E-F). In addition, we validated this in BV2 cells, where HK2 was immunoprecipitated, and OTUD1 was detected in the immunoprecipitate by immunoblotting, confirming the endogenous interaction between OTUD1 and HK2 (Fig. 7G).

### OTUD1 drives HK2 mitochondrial dissociation via Ala-rich domain binding and K63-specific deubiquitination

**Fig. 8:**
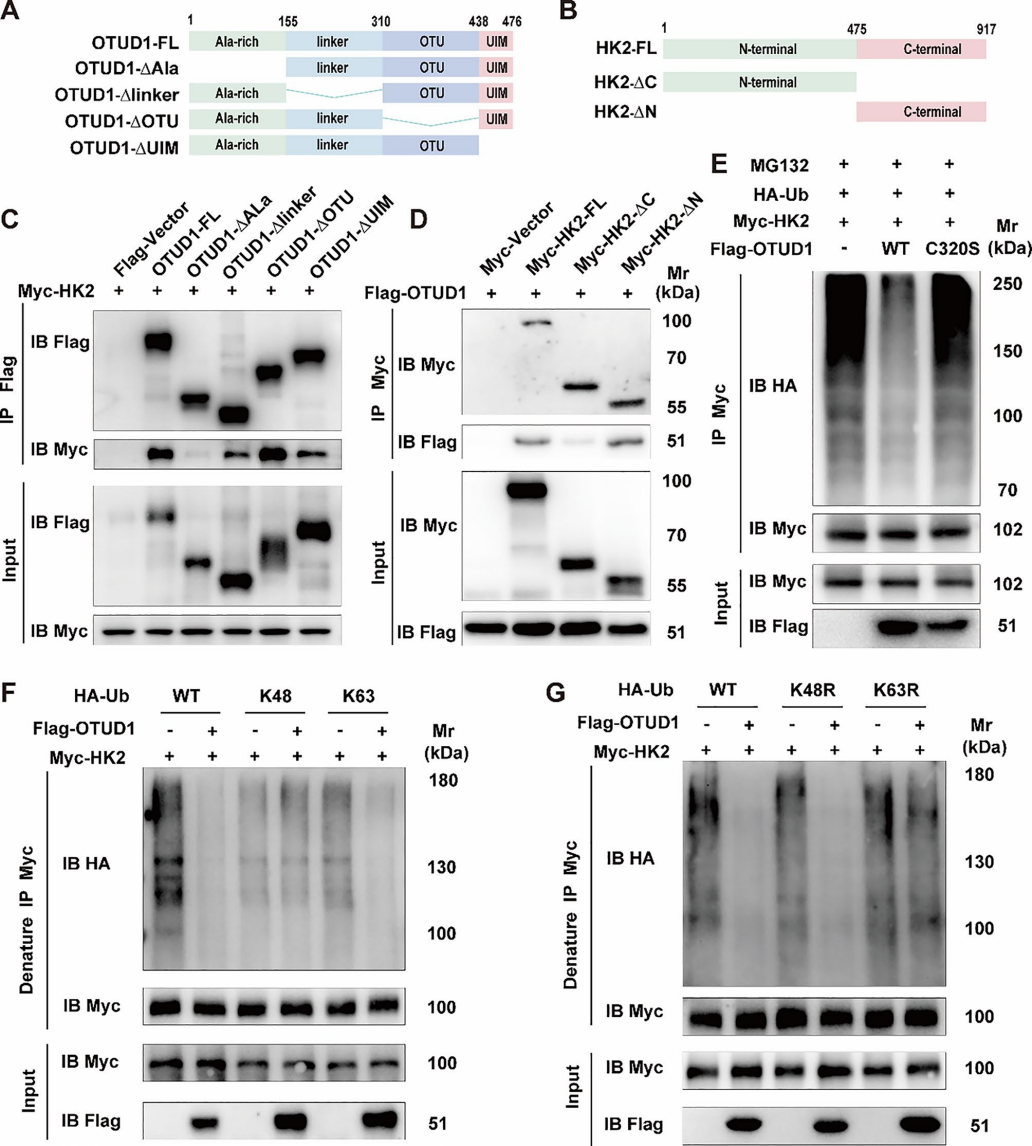
OTUD1 regulates the activity of HK2 protein through K63-linked ubiquitination. (**A-B**) Domain mapping of OTUD1 and HK2. (**C-D**) Transfection of truncated forms of OTUD1 and HK2 in 293T cells, followed by Co-IP to detect the interaction domains between OTUD1 and HK2 (*n* = 3/group). (**E**) In vitro assay of HK2 deubiquitination by OTUD1 and OTUD1 C320S (*n* = 3/group). (**F**) Transfection of Flag-OTUD1, Myc-HK2, HA-Ub, HA-K48 and HA-K63 into293T cells, followed by western blot to detect the ubiquitination of HK2 (*n* = 3/group). (**G**) Transfection of Flag-OTUD1, Myc-HK2, HA-Ub, HA-K48R and HA-K63R into 293T cells, followed by western blot to detect the ubiquitination of HK2 (*n* = 3/group).

To determine the particular domains involved in the interaction, we generated a series of truncation mutants (Fig. 8A-B) and found that the Ala-rich domain of OTUD1 plays a crucial role in its interaction with the C-terminal domain of HK2 (Fig. 8C-D). OTUD1 typically regulates various cellular processes through its deubiquitinase activity. Several studies have reported that the catalytically inactive OTUD1 mutant (C320S) is unable to exert its function by specifically removing ubiquitin molecules from target proteins [29, 30]. Here, we also found that OTUD1-C320S can no longer remove ubiquitins from HK2, indicating that OTUD1 deubiquitinates HK2 in a manner dependent on its enzymatic activity (Fig. 8E). Research has shown that the mitochondrial localization and function of HK2 depend on its K63-linked ubiquitination modification [31]. To identify which type of ubiquitin chain on HK2 is influenced by OTUD1, we conducted deubiquitination assays using K48 and K63 ubiquitin mutants. Our results demonstrated that OTUD1 specifically removed K63-linked ubiquitin chains from HK2 (Fig. 8F). To further demonstrate this result, we performed ubiquitination assays using the K48R and K63R ubiquitin mutant and found OTUD1 failed to reduce HK2 ubiquitination in the presence of K63R mutant ubiquitin (Fig. 8G). Therefore, we conclude that OTUD1 regulates the dissociation of HK2 from mitochondria by selectively removing K63-linked polyubiquitin chains from HK2. We subsequently examined the changes in K63 ubiquitination levels of HK2 following knockdown of OTUD1 in BV2 cells. As demonstrated in Figure S1G, LPS/nigericin stimulation significantly reduced the K63-linked ubiquitination level of HK2. However, this reduction was partially reversed upon OTUD1 knockdown under LPS/nigericin treatment. These data further support that OTUD1 facilitates HK2 dissociation from mitochondria and pyroptosis by reducing its K63 ubiquitination.

### OTUD1 is closely associated with human sepsis and 28-day survival rate

**Fig. 9:**
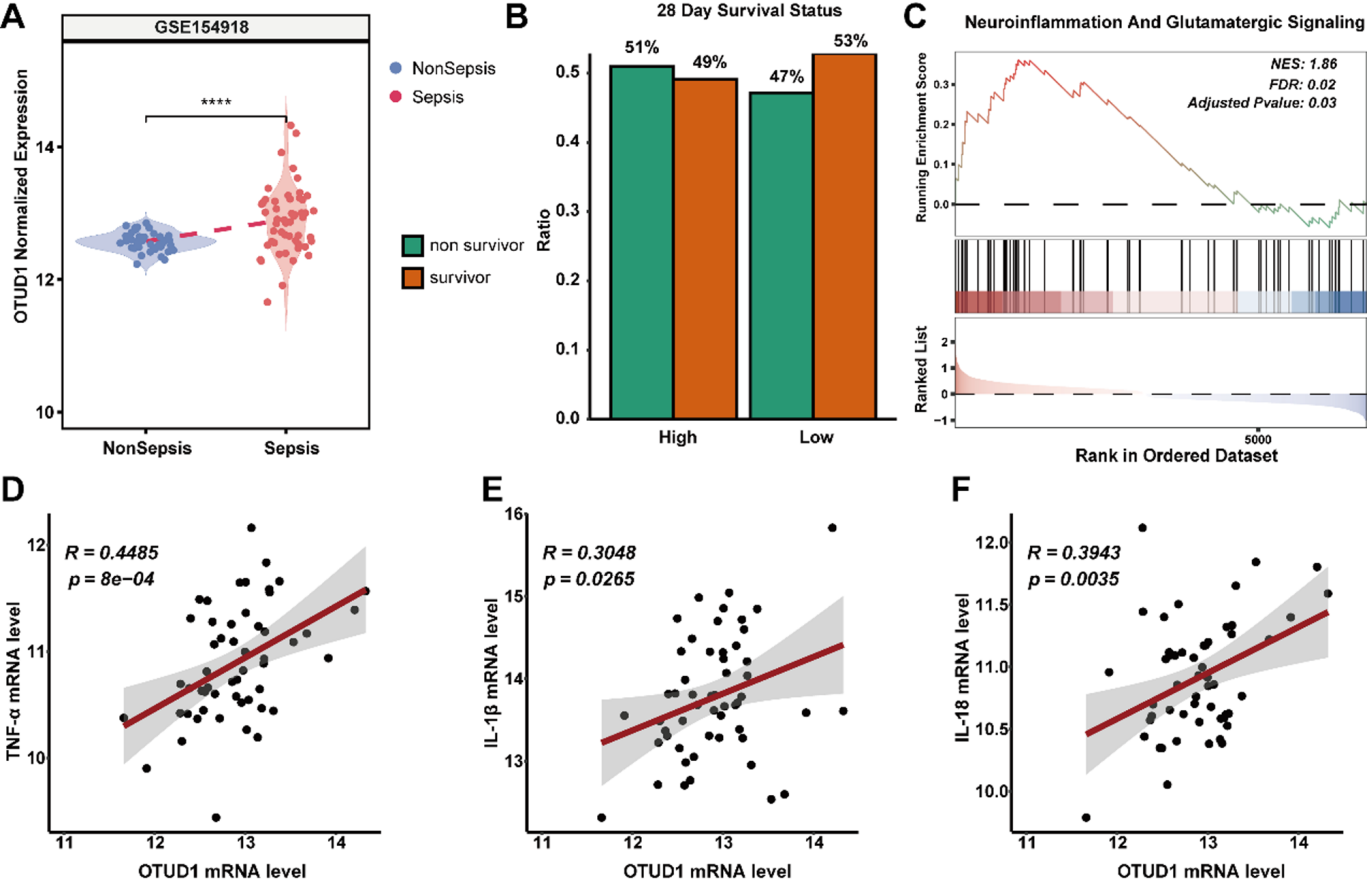
OTUD1 is closely associated with human sepsis and 28-day survival rate. (**A**) Expression levels of OTUD1 in sepsis patients compared to non-sepsis patients. (**B**) Relationship between OTUD1 expression levels and 28-day mortality in sepsis patients. (**C**) GSEA of high OTUD1 expression and low OTUD1 expression. (**D**-**F**) Pearson’s correlation analysis between OTUD1 and TNF-α, IL-1β, and IL-18 inflammatory factors.

To elucidate the clinical relevance of OTUD1 in sepsis and SAE, we interrogated publicly available sepsis datasets. Comparative analysis revealed significantly elevated OTUD1 expression levels in sepsis patients compared to healthy controls (GSE154918) (Fig. 9A). We further evaluated the prognostic value of OTUD1 by stratifying patients into high- and low-expression cohorts based on median OTUD1 mRNA levels. The results demonstrated that sepsis patients with lower OTUD1 expression exhibited a decreased 28-day mortality rate (53% vs. 47%) (Fig. 9B). Furthermore, Gene set enrichment analysis (GSEA) revealed that high OTUD1 expression was positively correlated with Neuroinflammation and Glutamatergic Signaling in sepsis patient (Fig. 9C), suggesting a potential link between OTUD1 and neurological dysregulation in sepsis. Additionally, Pearson’s correlation analyses revealed statistically significant positive associations between OTUD1 expression and circulating levels of pro-inflammatory cytokines, including TNF-α, IL-1β, and IL-18 (Fig. 9D-F). Collectively, these clinical and bioinformatic findings implicate OTUD1 as a critical regulator of neuroinflammation in sepsis pathophysiology, potentially mediated through microglial pyroptosis-dependent mechanisms.

## Discussion

**Fig. 10:**
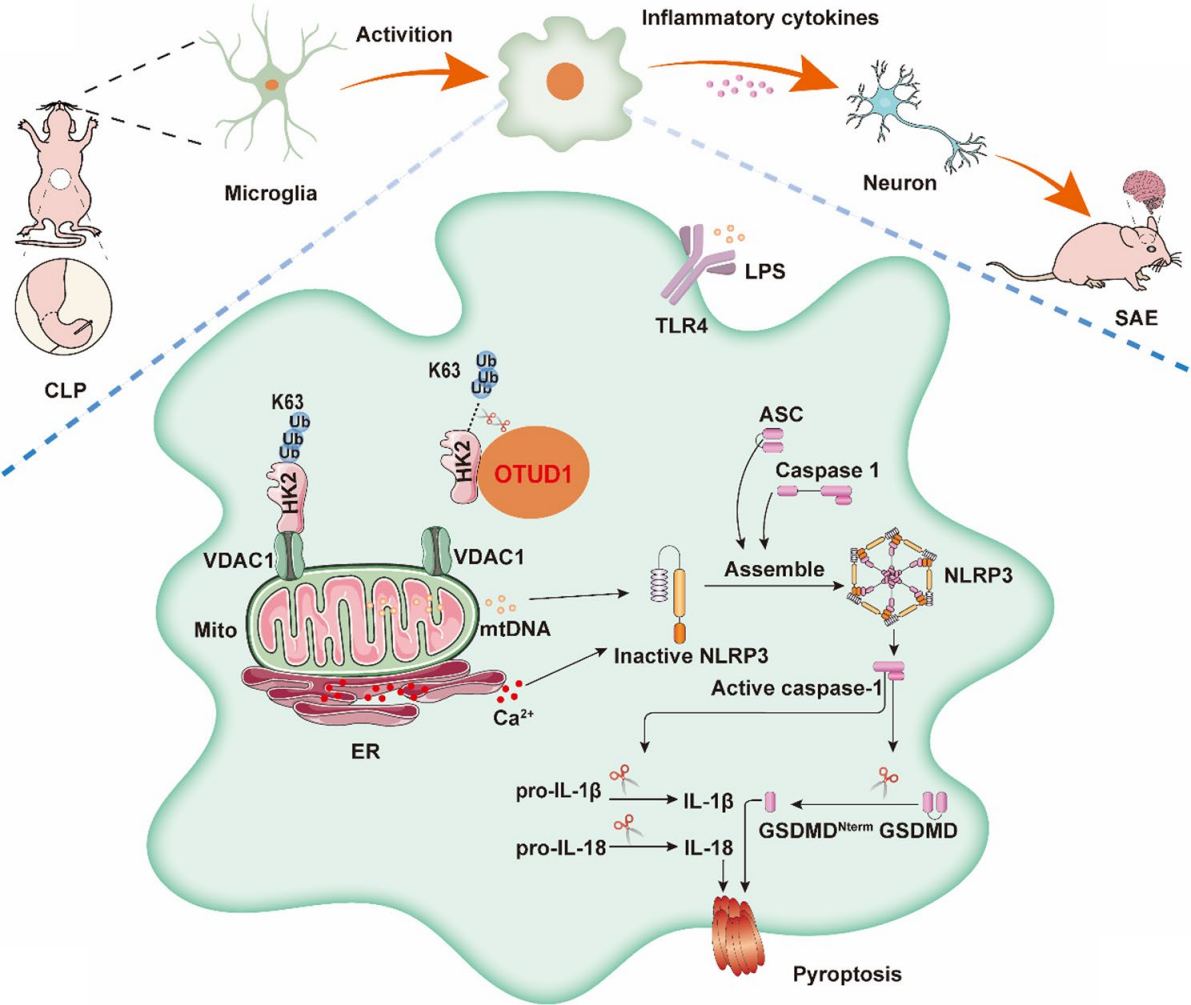
Mechanistic schematic illustrating OTUD1-mediated deubiquitination of HK2 in driving microglia pyroptosis during SAE.

SAE is a frequent and serious complication of sepsis, strongly linked to both mortality and morbidity. Microglia pyroptosis-mediated neuroinflammation is a key pathogenic mechanism of SAE [32]. In this study, we comprehensively identified the transcriptome of microglia in the hippocampus of CLP-induced SAE mice, revealing their progression from homeostatic clusters to an SAE-specific phenotype of microglia with OTUD1-dependent pro-pyroptotic activity in SAE. Further experiments demonstrated that OTUD1-mediated K63-linked ubiquitination on HK2 triggers mitochondrial HK2-VDAC1 dissociation, initiating VDAC1 oligomerization-mediated mtDNA release that activates NLRP3 inflammasome-driven microglia pyroptosis and pro-inflammatory cytokine release, ultimately leading to neuronal damage, synaptic dysfunction, and cognitive deficits in SAE, establishing OTUD1-regulated pyroptosis as a central pathogenic mechanism. (Fig. 10).

Recent advances in functional genomics have elucidated the spatial and temporal dynamics of microglial reactivity across diverse pathological contexts. Under neuropathological conditions, microglia characteristically downregulate homeostatic genes while upregulating a core transcriptional program defining the disease-associated microglia (DAM) phenotype - a reactive state specifically induced by tissue damage-associated stress signals. The DAM-like state has been observed in various disorders and is proposed as a conserved feature of microglial responses to CNS pathology [33–35]. Nevertheless, DAM-like microglia distinctly exhibit divergent molecular signatures and heterogeneous cellular kinetics in a context- and time-dependent manner. These findings highlight the necessity to investigate the complexities and variations of DAM-like microglia across different CNS pathologies. Studies have demonstrated that a microglial subpopulation associated with Alzheimer’s disease and amyotrophic lateral sclerosis contributes to restricting neurodegeneration [36]. These DAM elucidate the cellular heterogeneity and functional roles of immune cell subpopulations in these neurodegenerative disorders. We therefore conducted comparative analyses between DAM gene expression profiles/marker signatures and those of our newly identified SAM population. Our findings revealed that characteristic DAM-downregulated markers (including *P2ry12*, *Tmem119*, and *Gpr34*) were similarly downregulated in SAM, while DAM-upregulated markers (such as *Fth1*, *Cd63*, *Cd83*, *Cstb*, and *Apoe*) exhibited parallel upregulation in SAM. This transcriptional congruence suggests SAM may represent a DAM variant responsive to cerebral pathology. However, we observed SAM specifically expressed pyroptosis-related genes (*Nlrp3*, *Tnf*, and *Il1b*) at significantly higher levels, while DAM signatures emphasize phagocytosis (*Apoe*, *Trem2*) and lipid metabolism (*Fabp5*, *Lpl*). In addition, SAM exhibited marked upregulation of monocyte chemoattractants (*Ccl3*, *Ccl4*, and *Ccl5*) not prominent in DAM. This aligns with SAE’s systemic inflammatory milieu, where chemotaxis amplifies neuroinflammation. What’ more, SAM-specific TFs (*Nfkb2*, *Cebpb*, and *Foxp4*) drive pro-inflammatory and pyroptotic programs, differing from DAM-linked TFs (*Mafb*, *Spi1*). These differences demonstrate that SAM as a novel microglial subtype driving SAE progression via neuroinflammatory mechanisms. To our knowledge, this represents the first report demonstrating sepsis-induced microglial heterogeneity and identifying SAM as a unique pro-inflammatory microglial subset implicated in SAE pathogenesis.

Although DUBs extensively participate in the regulation of inflammatory responses, immune modulation, and cell death through multiple pathways in sepsis [37, 38]their role in SAE remains poorly understood. By analyzing the expression of 98 DUBs in different microglia subsets, we found that OTUD1 be closely related to the development of SAE. OTUD1 exerts different effects in various inflammatory diseases. In dextran sulfate sodium-induced colitis mice, OTUD1 binds to RIPK1, removing K63-linked polyubiquitination and inhibiting NF-κB activation, reducing pro-inflammatory cytokines and preventing colitis [27]. However, OTUD1 also deubiquitinates CARD9, promoting NF-κB activation in macrophages. Deleting OTUD1 in myeloid cells alleviates cardiac inflammation in mice [13]. Thus, OTUD1 has opposing roles in different inflammatory models. This may result from multiple factors, including cell type, microenvironment, signal pathway interactions, spatiotemporal regulation, and molecular modifications. Here, we found that OTUD1 promotes NLRP3 inflammasome activation and pyroptosis in microglia, contributing to the development and progression of SAE in mice. Clinical data also indicated that OTUD1 expression is obviously increased in sepsis patients, and higher levels of OTUD1 are highly correlated with neuroinflammation and 28-day mortality. This all indicates that OTUD1 may promote the pathophysiological progression of SAE by regulating microglia pyroptosis.

HK2, a pivotal glycolytic enzyme catalyzing glucose phosphorylation to generate glucose-6-phosphate, exhibits dual functional roles beyond energy metabolism, including the regulation of inflammatory cascades-notably NLRP3 inflammasome activation [39, 40]. Intriguingly, pharmacological inhibition of HK2-mediated glycolysis via 2-deoxyglucose paradoxically enhances NLRP3 inflammasome activation and IL-1β secretion, particularly under glucose-deprived conditions [41, 42]. Mechanistically, disruption of HK2-VDAC1 binding using the HKVBD peptide-which preserves HK2 catalytic activity while preventing mitochondrial association-induces dose-dependent IL-1β release in LPS-primed BMDMs [26]. These findings collectively indicate that physical dissociation of HK2 from mitochondria, rather than enzymatic inhibition, drives inflammasome activation. Following NLRP3 activation (e.g., ATP/nigericin stimulation), HK2 detachment from VDAC1 triggers IP3R-mediated ER Ca²⁺ efflux into mitochondria. This Ca²⁺ surge promotes VDAC1 oligomerization, forming mitochondrial outer membrane pores that facilitate mtDNA translocation to the cytosol. Cytosolic mtDNA subsequently recruits NLRP3 via VDAC1 oligomer interactions, initiating inflammasome assembly [11, 43]. In our experimental model, LPS/nigericin treatment induced mitochondrial HK2 dissociation, reduced ER Ca²⁺ stores, and elevated cytosolic mtDNA levels. Critically, OTUD1 knockout enhanced HK2-VDAC1 colocalization, normalized ER Ca²⁺ homeostasis, and attenuated cytosolic mtDNA accumulation. These data demonstrate that OTUD1 facilitates HK2 dissociation from mitochondria, thereby promoting NLRP3 inflammasome activation and pyroptosis.

K63-linked polyubiquitination serves as a critical regulatory mechanism governing the mitochondrial localization of HK2. The E3 ubiquitin ligase HectH9 mediates K63-linked ubiquitination of HK2, and genetic depletion of HectH9 destabilizes the HK2-VDAC1 interaction at mitochondrial membranes, ultimately triggering programmed cell death [31]. A pivotal gap in this regulatory axis lies in identifying a K63-specific DUB capable of counteracting HK2 ubiquitination to modulate its pro-inflammatory functions. Here, we demonstrate that OTUD1, a known hydrolase of K63-linked ubiquitin chains, directly interacts with the C-terminal domain of HK2 via its Ala-rich motif. Crucially, catalytic inactivation of OTUD1’s deubiquitinase domain (C320S mutation) abrogated its ability to cleave ubiquitin chains from HK2, confirming enzyme activity-dependent functionality. Further studies, whether in 293T cells or BV2 cells, demonstrate that OTUD1 selectively hydrolyzes K63-linked polyubiquitin chains on HK2, thereby reducing its mitochondrial localization. which is consistent with previous reports. This finding is mechanistically consistent with established literature documenting OTUD1’s enzymatic specificity toward cleavage of K63-linked ubiquitin chains.

However, some limitations in our study cannot be ignored. Firstly, OTUD1 global knockout mouse model employed in this study carries inherent limitations. A microglia-specific conditional KO model would provide superior precision in delineating OTUD1’s microglial functions by eliminating potential confounding effects from systemic ablation. However, our experimental design was rigorously supported by scRNA-seq and immunofluorescence data, which unequivocally demonstrated that OTUD1 is predominantly expressed in microglia, with minimal detection in other neural cell populations (e.g., neurons, astrocytes). This expression specificity indicates that the observed phenotypes in global KO mice primarily arise from microglial OTUD1 deficiency. To further validate cell-autonomous mechanisms, primary microglia isolated from OTUD1^−/−^ mice exhibited consistent pathological features with in vivo findings. Collectively, these data robustly support the conclusion that OTUD1 governs SAE through microglia-dependent pathways. Nevertheless, we acknowledge that conditional KO models remain critical for mechanistic refinement, and their development has been prioritized in subsequent studies to solidify the causal evidence chain. Additionally, while our truncation experiments identified the Ala-rich domain of OTUD1 and the C-terminal domain of HK2 as critical for their interaction, future structural studies are required to pinpoint specific binding residues. Such efforts will deepen our understanding of OTUD1-HK2 regulation in SAE.

In conclusion, we demonstrate that OTUD1 specifically cleaves K63-linked ubiquitination of HK2, thereby reducing its mitochondrial translocation, which promotes microglia pyroptosis and exacerbates cognitive dysfunction in SAE mice. Our data reveal the critical role of OTUD1 in regulating neuroinflammation in the CNS, suggesting that targeting OTUD1 may be a potential therapeutic approach for SAE. Pharmacological inhibition of OTUD1’s catalytic activity (via OTU domain-targeted compounds) could stabilize K63 ubiquitination on HK2, thereby preserving its mitochondrial retention and blocking pyroptosis. Clinically, elevated OTUD1 expression may serve as a biomarker to identify high-risk SAE patients for targeted therapy. Given OTUD1’s microglia-specific enrichment, nanoparticle-mediated CNS delivery could minimize off-target effects. Combination therapy with NLRP3 inhibitors may yield synergistic effects by dual blockade of pyroptosis pathways. We also highlight challenges: OTUD1’s anti-inflammatory roles in tissues like the intestine necessitate microglia-specific intervention strategies to avoid systemic impacts. Future studies will focus on validating safety using conditional knockout models and developing selective inhibitors based on the interaction between OTUD1 and HK2. Collectively, this study establishes a theoretical foundation and innovative strategies for SAE treatment from mechanistic to translational perspectives.

## Electronic supplementary material

Below is the link to the electronic supplementary material.

Supplementary Material 1

Supplementary Material 2

Supplementary Material 3

Supplementary Material 4

## Data Availability

No datasets were generated or analysed during the current study.

## Abbreviations

CLP: Cecal ligation and puncture
CNS: Central nervous system
Co-IP: Co-immunoprecipitation
DAM: Disease-associated microglia
DUBs: Deubiquitinating enzymes
ER: Endoplasmic reticulum
GSDMD: Gasdermin D
GSEA: Gene set enrichment analysis
HK2: Hexokinase 2
HM: Homeostatic microglia
mtDNA: Mitochondrial DNA
MWMT: Morris water maze test
NAM: Neural regulation-associated microglia
NORT: Novel object recognition test
OTUD1: Ovarian tumor deubiquitinase 1
PPI: Protein-protein interaction
PSD95: Postsynaptic density 95
RT-qPCR: Real-time quantitative polymerase chain reaction
SAE: Sepsis-associated encephalopathy
SAM: SAE-associated microglia
scRNA-seq: Single-cell RNA sequencing
snRNA-seq: Single-nucleus RNA sequencing
SYP: Synaptophysin
TEM: Transmission electron microscopy
TFs: Transcription factors
VDAC1: Voltage-dependent anion channel 1
WT: Wild-type
