# Supplementary material for: OTUD1 exacerbates sepsis-associated encephalopathy by promoting HK2 mitochondrial release to drive microglia pyroptosis
Source: J Neuroinflammation. 2025 Jun 11;22:154. doi: 10.1186/s12974-025-03480-w (PMC12153095; doi:10.1186/s12974-025-03480-w)
Supplement: Supplementary file 1 — Supplementary Material 1 [file 12974_2025_3480_MOESM1_ESM.docx]

**Figure S1**


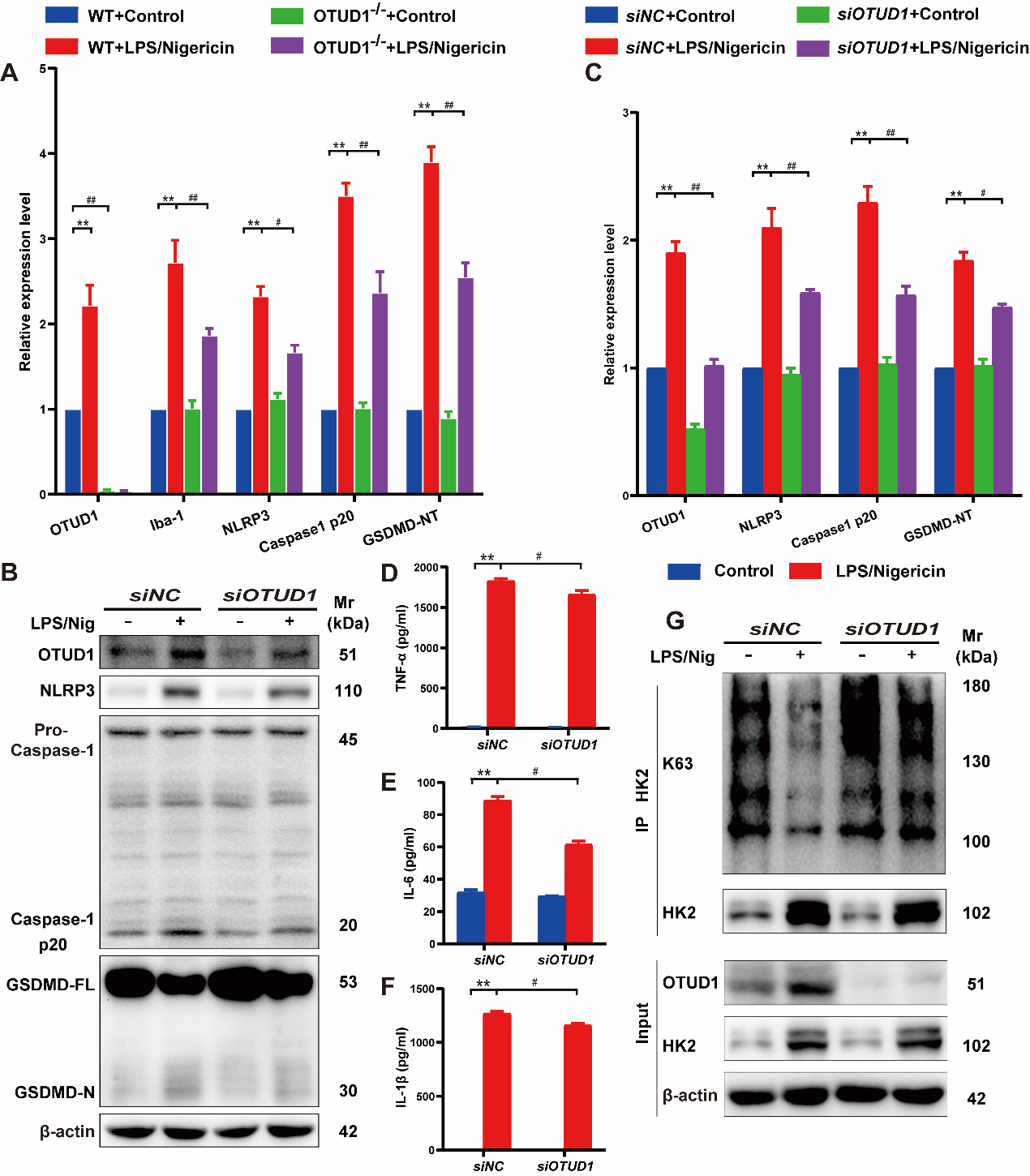


Figure S1 OTUD1 deficiency reduces NLRP3 inflammasome activation and pyroptosis of microglia in vitro. **(A)** The protein levels of OTUD1, Iba-1, NLRP3, Caspase-1 P20, and GSDMD-N in primary microglia were measured by western blot **(B-C)** The protein levels of OTUD1, NLRP3, Caspase-1 P20, and GSDMD-N in BV2 cells were measured by western blot (n=3/group). **(D-F)** TNF-α, IL-6 and IL-1β in the culture supernatant of BV2 cells were measured by ELISA (n=3/group). **(G)** Immunoblot analysis of HK2 K63-linked ubiquitination in BV2 cells transfected with negative control siRNA and OTUD1 siRNA following LPS/Nigericin stimulation (n=3/group). ***p<*0.01 vs. Control group; ^##^*p*<0.01 vs. LPS/Nigericin group.
